# Lower academic performance among children with perinatal HIV exposure in Botswana

**DOI:** 10.1002/jia2.26165

**Published:** 2023-11-01

**Authors:** Kathleen M. Powis, Lesedi Lebanna, Sara Schenkel, Gosego Masasa, Samuel W. Kgole, Martha Ngwaca, Coulson Kgathi, Paige L. Williams, Amy L. Slogrove, Roger L. Shapiro, Shahin Lockman, Mompati O. Mmalane, Joseph M. Makhema, Jennifer Jao, Adam R. Cassidy

**Affiliations:** ^1^ Department of Internal Medicine and Pediatrics Massachusetts General Hospital Boston Massachusetts USA; ^2^ Department of Immunology and Infectious Diseases Harvard T.H. Chan School of Public Health Boston Massachusetts USA; ^3^ Botswana Harvard Health Partnership Gaborone Botswana; ^4^ Department of Curriculum Development and Evaluation Botswana Ministry of Basic Education Gaborone Botswana; ^5^ Departments of Biostatistics and Epidemiology Harvard T.H. Chan School of Public Health Boston Massachusetts USA; ^6^ Department of Paediatrics & Child Health Faculty of Medicine and Health Sciences Stellenbosch University Worcester South Africa; ^7^ Division of Infectious Diseases Brigham and Women's Hospital Boston Massachusetts USA; ^8^ Department of Pediatrics Northwestern Feinberg School of Medicine Chicago Illinois USA; ^9^ Departments of Psychiatry & Psychology and Pediatric & Adolescent Medicine Mayo Clinic Rochester Minnesota USA

**Keywords:** academic performance, Botswana, children, HIV‐exposed uninfected, neurodevelopment, primary school

## Abstract

**Introduction:**

Studies have reported a higher risk of suboptimal neurodevelopment among children who are HIV‐exposed uninfected (HEU) compared to children HIV‐unexposed uninfected (HUU). Actual academic performance among school‐aged children by HIV exposure status has not been studied.

**Methods:**

Academic performance in Mathematics, Science, English, Setswana and overall among children enrolled in the Botswana‐based FLOURISH study who were attending public primary school and ranging in age from 7.1 to 14.6 years were compared by HIV exposure status using a Cochran‐Mantel‐Haenszel test. Lower academic performance was defined as a grade of “C” or lower (≤60%). Unadjusted and adjusted logistic regression models were fit to assess for an association between HIV exposure and lower academic performance.

**Results:**

Between April 2021 and December 2022, 398 children attending public primary school enrolled in the FLOURSH study, 307 (77%) were HEU. Median age was 9.4 years (IQR 8.9–10.2). Only 17.9% of children HEU were breastfeed versus 100% of children HUU. Among children HEU, 80.3% had foetal exposure to three‐drug antiretroviral treatment, 18.7% to zidovudine only and 1.0% had no antiretroviral exposure. Caregivers of children HEU were older compared to caregivers of children HUU (median 42 vs. 36 years) and more likely to have no or primary education only (15.0% vs. 1.1%). In unadjusted analyses, children HEU were more likely to have lower overall academic performance compared to their children HUU (odds ratio [OR]: 1.96 [95% confidence interval (CI): 1.16, 3.30]), and lower performance in Mathematics, Science and English. The association was attenuated after adjustment for maternal education, caregiver income, breastfeeding, low birth weight and child sex (aOR: 1.86 [95% CI: 0.78, 4.43]).

**Conclusions:**

In this Botswana‐based cohort, primary school academic performance was lower among children HEU compared to children HUU. Biological and socio‐demographic factors, including child sex, appear to contribute to this difference. Further research is needed to identify modifiable contributors, develop screening tools to identify the risk of poor academic performance and design interventions to mitigate risk.

## 1 INTRODUCTION

As the HIV epidemic has matured globally, successful scale‐up and access to three‐drug antiretroviral treatment (ART) among pregnant and breastfeeding persons living with HIV (PLHIV) has dramatically reduced the rate of infant HIV acquisition, from 24% of births in 2010 to 12% in 2021 globally, with high burden settings achieving rates of under 4%, including Botswana, Eswatini and South Africa [1]. Despite laudable gains towards eliminating infant HIV acquisition, the number of infants born to PLHIV has not changed substantially in the last decade, with over one million infants born annually with perinatal HIV exposure [1]. UNAIDS estimates the population of children under 15 years‐of‐age born HIV‐exposed uninfected to be 16 million in 2022, while the population of similarly aged children living with HIV was estimated at 1.5 million [1].

Starting life HIV‐free has not ensured that children with perinatal HIV exposure who remain uninfected achieve comparable health, growth or neurodevelopment outcomes compared to children born in the absence of perinatal HIV exposure. While findings are inconsistent, many studies report a higher risk of infectious morbidity and mortality, poorer growth outcomes and developmental delays among children HIV‐exposed uninfected (HEU) compared to children HIV‐unexposed uninfected (HUU) [2, 3, 4, 5, 6, 7, 8, 9, 10, 11, 12, 13]. The aetiology of this higher risk among children HEU is multifactorial, encompassing both biological and social determinants of health, including altered immunity early in life [14, 15, 16, 17, 18], a proinflammatory state in infancy [19, 20, 21], higher risk of preterm birth [22, 23], suboptimal duration of breastfeeding [3, 24], poor maternal health [25] and household food insecurity [26].

Biological and socio‐demographic factors have been associated with neurodevelopmental delays in children, regardless of their HIV exposure status [27, 28, 29, 30, 31, 32, 33]. However published research from high HIV burden settings employing a variety of neurodevelopmental assessment tools has reported a higher risk of poorer neurodevelopmental outcomes, including poorer neurocognitive functioning [11, 34], gross motor delays [34, 35] and lagging language skills [34, 35] among children HEU compared to children HUU. The collective body of published work on neurodevelopmental outcomes of children HEU has relied on neurodiagnostic testing to assess for differences between children HEU and those who are HUU. While testing results offer important data, actual academic performance has been closely linked to a person's physical and mental health and their ability to contribute productively to society [36]. However, actual academic performance has not specifically been evaluated in children who are HEU compared to those who are HUU.

Using data from a prospective birth cohort study in Botswana, the FLOURISH (**F** ollowing **L** ongitudinal **O** utcomes to **U** nderstand, **R** eport, **I** ntervene and **S** ustain **H** ealth Outcomes for Infants, Children, and Adolescents who are HIV‐Exposed Uninfected) study, we sought to evaluate differences in actual academic performance by child HIV exposure status in a subset of children attending public primary school where curriculum is standardised nationally, as are testing, and grading.

## 2 METHODS

### 2.1 Study population, design and ethical considerations

The FLOURISH study is an ongoing prospective observational Botswana‐based study being conducted by Botswana Harvard Health Partnership (BHP). Children HEU and those HUU are being recruited after previous participation in a BHP birth cohort study, including the Mma Bana [37], Mpepu [38] and Tshipidi [39] studies, all of which have been previously described. The Mma Bana study enrolled treatment naïve pregnant PLHIV with CD4 cell counts ≥200 cells/mm^3^, randomizing participants to one of two ART regimens between 26‐ and 34‐weeks gestation, as well as pregnant PLHIV with CD4 cell counts <200 cells/mm^3^ who were between 18‐ and 34‐weeks gestation and already receiving the first‐line ART regimen per Botswana national treatment guidelines [37]. The Mpepu study, investigating any potential survival benefit with long‐term use of cotrimoxazole for infants HEU, allowed for study enrolment in pregnancy or as late as the infant's 34th day of life, as infants were not randomised to cotrimoxazole or placebo until between 30 and 34 days of life under the original study protocol [38]. The Tshipidi study enrolled both PLHIV and those who were seronegative during pregnancy or within 7 days of the infant's birth [39].

Since the curriculum in Botswana public schools is standardised by the Department of Curriculum Development within Botswana's Ministry of Education and Skills Development, and examinations are standardised by the Botswana Examinations Council, as are grading criteria, the academic performance of children attending public schools was analysed overall and in the subjects of Mathematics, Science, English and Setswana, the native language of Botswana. Children who attended private schools were excluded from the analysis, as private schools in Botswana are not required to abide by requirements established by Botswana's Department of Curriculum Development or the Botswana Examinations Council. Additionally, children participating in special education were excluded. If a mother enrolled in a prior BHP study was HIV uninfected during the previous study but subsequently acquired HIV prior to enrolment in the FLOURISH study, the child was excluded from the academic performance analysis. While the FLOURISH study recruits children and adolescents of all ages from prior BHP studies, fewer prior BHP studies involved enrolment of children HUU. Therefore, in order to have an appropriately sized comparator group, only grade levels where at least 15% of FLOURISH participants were children HUU were included in the analysis, restricting the analysis to participants attending primary education grades designated as standard 3 through 6, where annual progression would be expected to have children 7 years‐of‐age through 12 years‐of‐age in these grades, depending on a child's date of birth at the start of a school year. All adult participants provided written informed consent, on their own behalf and that of their child's, for study participation. For children ≥7 years of age, assent is also obtained from the child. The FLOURISH study protocol was approved by the Botswana Health Research and Development Committee, as was well Mass General Brigham Institution Review Board.

### 2.2 Confirmation of HIV status

All biological mothers not known to be living with HIV at the completion of the prior BHP study in which they participated, and all children participating in the FLOURISH study undergo HIV testing and counselling upon enrolment into the FLOURISH study to confirm HIV status as a condition of study enrolment. Only pairs where the child has a negative HIV test are eligible for continued participation in the FLOURISH study.

### 2.3 Historical maternal‐child database

A harmonised maternal‐child database exists, housing common data elements for key historical BHP maternal‐child research studies, including the studies from which the FLOURISH study is recruiting. Maternal data elements include but are not limited to HIV status, date of enrolment, timing of enrolment, either in pregnancy or within 34 days of giving birth, age at enrolment, obstetric history and socio‐demographic information at time of enrolment. For women living with HIV at the time of the prior BHP study, if originally collected, the database includes CD4 cell count and HIV‐1 viral load at time of enrolment, either during pregnancy or within 34 days of giving birth, timing of ART initiation, either prior to conception or during pregnancy, date of ART initiation (if known) and ART taken in pregnancy as none, zidovudine (AZT) only or a three‐drug ART regimen. Harmonised child data, if originally collected, includes gestational age at birth, sex, and infant feeding mode (breast vs. formula). Children who participated in the Mma Bana, Mpepu and Tshipidi studies are now of school age and the historically collected maternal‐child data has been used in this analysis of academic performance.

### 2.4 Primary outcome—academic performance

At enrolment, grades from the child's latest school report card are abstracted, including subject grades and the child's overall grade. Academic performance was assessed for grades in Mathematics, Science, English, Setswana and the overall grade. Lower academic performance was defined as a grade of C or lower (≤60%). Academic performance was analysed for eligible children enrolled in the FLOURISH study between 30 April 2021 and 31 December 2022.

### 2.5 Exposures of interest

The primary exposure of interest was foetal HIV exposure. Other potential exposures associated with childhood neurodevelopment with implications for school performance evaluated as potential predictors of academic performance included child preterm birth (<37 weeks completed gestational age), child sex and ever being breastfed (variables abstracted from the maternal‐child database); and maternal education level, maternal positive screen for depression or anxiety (using the Patient Health Questionnaire [PHQ9] and General Anxiety Disorder‐7 [GAD7] screening tools) and proxies for household poverty, including caregiver reported income, absence of electricity in the home and report of household food insecurity in the last 12 months (collected in the FLOURISH study), with the latter two assumed, a priori, to be associated with academic performance, as well [32, 40–42].

### 2.6 Statistical analysis

Baseline caregiver and child characteristics were compared by child HIV exposure status using Wilcoxon Rank Sum tests for continuous variables and Chi‐squared or Fisher's exact tests for categorical or ordinal variables. School grades, by subject and overall, were dichotomised with grades of A or B categorised as higher academic performance and grades of C or lower categorised as lower academic performance. A Cochran‐Mantel‐Haenszel test was used to compare academic performance by HIV exposure status, by subject and overall. Unadjusted and adjusted logistic regression models were fit to assess the association between HIV exposure status and lower overall academic performance. Covariates in unadjusted models with a *p*‐value of ≤0.20 were included in the adjusted model. Logistic regression models were also fit to assess associations between maternal HIV disease status and treatment with overall academic performance in the subset of children HEU. Analyses were performed with SAS, version 9.4 (SAS Institute, Inc).

## 3 RESULTS

Between 30 April 2021 and 31 December 2022, 413 children ranging in age from 7.1 to 14.6 years were enrolled in the FLOURISH study and were attending standard 3 through standard 6 primary education. Eight children were attending private school, including five (1.2%) who were HEU and three (3.0%) who were HUU, and these eight were excluded from the analysis of public school academic achievement. Another four children were enrolled in the FLOURISH study with a mother who was HIV uninfected at the time of participation in the original BHP study, but since the biological mother had subsequently acquired HIV prior to enrolment in the FLOURISH study, these children were excluded from the academic achievement analysis. Finally, three children, including two HEU, were participating in special education curriculum. The analysis of academic performance among FLOURISH study participants attending standard 3 through standard 6 includes 398 children, of whom 373 (94.7%) were of appropriate age for grade level, while the remainder 25 (6.3%) were older than what would be expected for grade level, including 20 (6.5%) children who were HEU and five (5.5%) who were HUU.

The characteristics of caregivers and children are presented in Table 1. Caregivers of children HEU were older on average compared to caregivers of children HUU (median age 41.9 vs. 36.0 years), with 97% of all caregivers, regardless of a child's HIV exposure status, being the biological mother of the child. Caregivers of children HEU more often had no formal education or had a primary school education only and less often had completed tertiary education than caregivers of children HUU. A higher proportion of caregivers of children HEU were married or cohabiting compared to caregivers of children HUU. Few children HEU had ever breastfed, while 100% of children HUU had been breastfed. Children HEU were also younger than children HUU (median 9.3 years‐of‐age vs. 9.9).

**Table 1:** Caregiver and child characteristics by child HIV exposure status

Maternal characteristics	Caregivers of children HEU (*n* = 307)	Caregivers of children HUU (*n* = 91)	*p*‐value
Median caregiver age at enrolment (IQR)	41.9 (37.0–45.7)	36.0 (32.8–41.5)	<0.0001
Highest maternal education level			<0.0001
No or primary school	46 (15.0%)	1 (1.1%)	
Junior or senior secondary school	232 (75.6%)	63 (70.0%)	
Tertiary school	29 (9.4%)	29 (28.9%)	
Marital status # (%)			0.05
Single	168 (54.7%)	61 (68.5%)	
Married/co‐habiting	125 (40.7%)	28 (31.5%)	
Divorced	5 (1.7%)	0 (NA)	
Widowed	9 (2.9%)	0 (NA)	
Maternal monthly income			0.002
None	91 (30.5%)	25 (28.1%)	
$1–$50	33 (11.1%)	10 (11.2%)	
$51–$100	51 (17.1%)	9 (10.1%)	
$101–$500	110 (36.9%)	30 (33.7%)	
>$500	14 (4.4%)	16 (16.9%)	
Electricity in home # (%)	269 (87.6%)	81 (89.0%)	0.86
Household food insecurity reported in the last 12 months prior to enrolment # (%)	100 (33.1%)	22 (24.4%)	0.15
Biological mother as caregiver # (%)	297 (96.7%)	90 (98.9%)	0.47
Original BHP study			<0.0001
Mma Bana	5 (1.6%)	0 (NA)	
Mpepu	241 (78.5%)	0 (NA)	
Tshipidi	61 (19.9%)	91 (100%)	
Community			<0.0001
Gaborone (city)	182 (59.3%)	48 (52.8%)	
Lobatse (town)	26 (8.5%)	0 (NA)	
Mochudi (village)	27 (8.8%)	43 (47.2%)	
Molepolole (village)	72 (23.5%)	0 (NA)	
ARV treatment/prophylaxis in pregnancy # (%)			NA
None	3 (1.0%)	NA	
Zidovudine only	56 (18.7%)	NA	
Three‐drug antiretroviral treatment regimen	240 (80.3%)	NA	
Median CD4^+^ cell count at enrolment in original study	475 (353–626)	NA	NA
Virally suppressed at enrolment in original study # (%)	94 (40.9%)	NA	NA
Median viral load at enrolment in original study for participants with unsuppressed viral load (IQR)	4781 (390–25,539)	NA	NA

**Child characteristics**	HEU (*n* = **307**)	HUU (*n* = **91**)	
Median age of school‐age children in years	9.3 (8.8–10.1)	9.9 (9.2–10.4)	0.001
Male # (%)	155 (50.5%)	52 (57.1%)	0.28
Preterm # (%)	37 (12.3%)	10 (11.0%)	0.85
Low birth weight (≤2500 grams)	58 (18.9%)	6 (6.7%)	0.005
Ever breastfed # (%)	55 (17.9%)	91 (100%)	<0.0001

*Note*: Missing variables: Highest educational level—one caregiver of a child HUU; Marital status—two caregivers of children HUU; Income: nine caregivers of children HEU and two caregivers of children HUU; Household food insecurity—five missing for households of children HEU and one missing from household of a child HUU; Antiretroviral treatment/prophylaxis in pregnancy; eight caregivers of children HEU; CD4^+^ cell count: two for caregivers of children HEU; Viral load: 77 for caregivers of children HEU; Preterm: five missing for children HEU; Low birth weight—one for a child HUU.

Abbreviations: BHP, Botswana Harvard Health Institute Partnership; HEU, HIV‐exposed uninfected; HUU, HIV‐unexposed uninfected; IQR, interquartile range; NA, not applicable.

Children HEU had higher odds of lower academic performance in Science (2.23 [95% confidence interval (CI) 1.31, 3.80]), Mathematics (1.73 [95% CI 1.03, 2.93]), English (1.83 [95% CI 1.09, 3.08]) and overall (1.96 [95% CI 1.16, 3.30]) compared to children HUU. For Setswana, academic performance did not differ significantly by HIV exposure status (1.25 [95% CI 0.75, 2.10]).

In unadjusted analysis, child HIV exposure status was significantly associated with an increased risk of lower overall academic performance, as was an absence of maternal formal education or highest level as primary school compared to secondary or tertiary education, and male sex of the child (Table 2). The presence of caregiver depression or anxiety and the three proxy markers of poverty, including household income, absence of household electricity or food insecurity in the last year, were not significantly associated with a higher risk of lower overall academic performance. After adjustment for low maternal education, caregiver income, sex at birth, low birth weight and breastfeeding status, the association between HIV exposure and academic performance was attenuated, (adjusted OR = 1.86, 95% CI 0.78, 4.43), with male sex being the only significant predictor of lower overall academic performance.

**Table 2:** Logistic regression model of factors associated with lower academic performance

	Unadjusted models		Adjusted model	
Covariates of interest	Odds ratio (95% CI)	*p*‐value	Odds ratio (95% CI)	*p*‐value
HEU versus HUU	1.96 (1.16, 3.30)	0.01	1.86 (0.78, 4.43)	0.16
Low maternal education^a^	2.40 (1.03, 5.60)	0.04	1.86 (0.77, 4.49)	0.17
Caregiver depression/anxiety at enrolment^b^	1.07 (0.49, 2.32)	0.87		
No caregiver income or income ≤$100 USD per month	1.75 (1.09, 2.81)	0.02	1.57 (0.96, 2.56)	0.07
Absence of household electricity	1.48 (0.70, 3.14)	0.31		
Household food insecurity in the last year^c^	0.82 (0.50, 1.36)	0.44		
Male child	1.80 (1.12, 2.89)	0.02	1.92 (1.17, 3.16)	0.01
Preterm birth^d^	1.43 (0.67, 3.03)	0.36		
Low birthweight (<2500 grams)	1.71 (0.84, 3.50)	0.14	1.61 (0.77, 3.367)	0.21
Never breastfed	1.38 (0.86, 2.22)	0.19	0.82 (0.37, 1.79)	0.61

*Note*: Covariates in unadjusted analyses with a *p*‐value ≤0.20 were included in the adjusted model.

Abbreviations: HEU, HIV‐exposed uninfected; HUU, HIV‐unexposed uninfected; CI, confidence interval; USD, United States Dollar.

^a^ Low maternal education was defined as no or primary education only.

^b^ Maternal depression was evaluated using the PHQ9 screening tool and anxiety via the GAD7 screening tool.

^c^ Household food insecurity was defined as being present if a caregiver reported that in the last 12 months the household ever had to cut the size of meals or skip meals because there was not enough food in the household.

^d^ Preterm birth was defined as a birth before 37.0 weeks completed gestational age.

Maternal HIV‐related covariates were evaluated to assess for association with overall lower academic achievement only among the 307 children HEU. Using data collected from the biological mother during the original BHP study, enrolment CD4^+^ cell count (OR 0.75 [95% CI 0.13, 4.18]), detectable pregnancy viral load (OR 1.06 [95% CI 0.54, 2.06]) and absence of receiving a three‐drug ART regimen in pregnancy (OR 0.88 [95% CI 0.44, 1.77]) were not found to be associated with lower academic performance.

## 4 DISCUSSION

In an analysis of actual academic performance among FLOURISH participants attending public primary school, children HEU were more likely to have lower academic performance in Mathematics, Science, English and overall compared to children HUU. However, in adjusted analysis, the association between HIV exposure status and academic performance was attenuated and only male child was associated with lower academic performance. The fact that a significant statistical association was not found between HIV exposure status and academic performance may reflect inadequate power to detect a true association. Larger studies of actual academic performance would be beneficial. Interestingly, when the covariate breastfeeding was removed from the model, HIV exposure was noted to be significantly associated with lower academic performance, highlighting the known protective effect of breastfeeding on neurodevelopment [29]. Among children HEU, maternal CD4^+^ cell count <200 cells/mm^3^, detectable viral load at the time of entry in the original BHP study, and absence of receiving a three‐drug ART regimen in pregnancy, three potentially biological etiologies, were not significantly associated with lower overall academic performance. This is the first study to compare actual academic performance between school‐age children by HIV exposure status. This is an important approach, as performance in school, from childhood through young adulthood, has been shown to be closely associated with a person's physical and mental health, as well as their ability to successfully integrate into society [36].

Various studies have been published on cognitive outcomes, comparing children who are HEU to those who are HUU, with mixed findings. Benki‐Nugent and colleagues evaluated neurocognitive functioning in a cohort of Kenyan children ages 5–12 years, including 56 children HEU and 65 HUU, employing multiple standardised neuropsychological tests [11]. Children HEU had significantly lower mean z‐scores for global cognitive ability, short‐term memory, attention and processing speed after adjusting for child age and sex, caregiver age, caregiver education, child nutritional status, household food security and orphanhood [11]. In a Canadian cohort, early academic achievement was measured utilizing the Word Reading, Spelling, and Math Computation subtests of the Wide Range Achievement Test—Fourth Edition (WRAT4) [43] among 110 children HEU and 43 children HUU. While children HEU scored within the average range on these three measures, mean scores were significantly lower compared to children HUU in word reading and math computation [12]. Interestingly, the Tshipidi study, the BHP study in which a portion of our FLOURISH caregiver‐child pairs participated, found no difference in neurodevelopmental outcomes at 24 months of life between children HEU and those who were HUU [39]. Our study, although not relying on a standardised battery of neurocognitive testing instruments, provides a pragmatic approach to assessing academic achievement. Participants in the FLOURISH study live in the same communities, regardless of their HIV exposure status. The curriculum is standardised nationally, developed by the Department of Curriculum Development within Botswana's Ministry of Education and Skills Development. Additionally, testing by subject matter is standardised by the Botswana Examinations Council, which also has established national standards for grading of test results. Although prior published studies comparing neurodevelopmental outcomes between children HEU and those who are HUU have relied on a battery of standardised testing instruments [35, 44, 45], it is critically important to evaluate actual academic performance, as this can be expected to exert a more direct influence on a child's self‐esteem, self‐confidence and enthusiasm for learning.

There are many strengths of the FLOURISH study, and some limitations as well. FLOURISH study participants being re‐enrolled after historical participation in birth cohort studies conducted by BHP have allowed us to leverage detailed prospectively collected data on their mother's pregnancy and their health, feeding practices and socio‐demographic history. The study design minimises misclassification of a child's HIV exposure status in infancy and their current HIV status. Participants reside in the same communities. Additionally, the fact that the curriculum in public schools in Botswana is standardised by grade level and subject, as is testing and grading is a major strength of this study. However, as in any observational study, there is the potential for unmeasured confounders. For example, data on maternal alcohol and substance use in pregnancy, although rare, were not collected historically. Given the cross‐sectional design of this study, with grades collected at study enrolment, some of the children's grades would have reflected an entire school year, while others may have only represented the first semester. While it is possible that a lower grade in the first semester could motivate a student, caregivers or a teacher to intervene, the FLOURISH data highlight the fact that children HEU were disproportionately more likely to have lower grades in key subjects and overall. The FLOURISH study has enrolled fewer children HUU and, for this analysis, all were from a single prior BHP study, Tshipidi. Tshipidi children were recruited from two of the four communities from which FLOURISH participants were recruited, Gaborone and Mochudi. In a separate sensitivity analysis restricted to children recruited from Gaborone and Mochudi only, the findings were similar, both for individual subjects and overall academic performance. It is challenging to disentangle the association between breastfeeding and academic performance, given that 100% of children HUU were breastfed, while only 18% of children HEU ever breastfed. Unfortunately, we do not have historical data collected on the duration of breastfeeding for all participants. Further research is needed to understand associations between the duration of exclusive and overall breastfeeding and academic performance. In this analysis, we selected household income, report of food insecurity in the last 12 months and absence of electricity in the home as markers of poverty, with the latter two independently associated with lower academic performance [32, 40–42]. In unadjusted analysis, only household income was associated with lower academic performance and there was a high prevalence of households with electricity at 87.9%. Future studies should explore the most significant markers of poverty associated with academic performance, as poverty eradication interventions could be designed, tested and implemented to improve academic performance. Finally, we recognise that maternal and family adjustment to a diagnosis of HIV, maternal disclosure to others, including children, and the support received or stigma experienced by a person living with HIV can influence a child's academic performance. We used measures of caregiver mental health, specifically screening instruments for depression, PHQ9, and anxiety, GAD7, as surrogates for maternal adjustment to living with HIV. We acknowledge that this may not have fully captured the impact of a caregiver's experience of living with HIV on a child's academic performance.

Suboptimal neurodevelopmental outcomes, which impact actual academic performance, are influenced by individual, family and societal factors. In this quantitative analysis, societal factors were not measured and limited data were collected on potential familial factors. Future studies of actual academic performance among children HEU would benefit from a multi‐level mixed methods study design measuring factors, such as illness of parents or siblings, school absenteeism, bullying, stigma, household internet access and contextually appropriate structural factors, at a minimum. Incorporation of qualitative in‐depth interviews structured to capture the impact of individual family dynamics over time which could influence a child's academic performance would also be beneficial.

## 5 CONCLUSIONS

In summary, there are a host of psychosocial factors that can influence a child's academic performance. While children who are HEU participating in the Botswana‐based FLOURISH study were more likely to have lower academic performance in Mathematics, Science, English and in their overall grade compared to children HUU, only being a male child was significantly associated with observed differences in adjusted analyses. In this cohort, children HEU were significantly less likely to ever have been breastfed, an activity with proven neurodevelopmental benefits. As this is the first study to pragmatically compare academic performance by child HIV exposure status, additional studies are needed to validate our findings. It will be important to identify risk factors amenable to interventions. Additionally, given the large and expanding population of children HEU, screening tools are urgently needed to identify children most at risk for poor academic achievement.

## COMPETING INTERESTS

No authors have competing interests or have conflict of interest against the work being performed in the FLOURISH study.

## AUTHORS’ CONTRIBUTIONS

KMP conceived of this study, performed the statistical analysis and drafted the initial manuscript. SS prepared the datasets for the analysis. SWK, MM and MN performed data cleaning on anomalous data. PLW and ALS provided guidance on the statistical analysis plan and interpretation. LL, SS, GM, SWK, MN, CK, PLW, ALS, RLS, SL, MOM, JMM, JJ and ARC all provided editorial input in the preparation of the final manuscript.

## FUNDING

The FLOURISH study is funded by the Eunice Kennedy Shriver National Institute of Child Health and Human Development (R33 HD103099; Principal Investigators: Powis, Jao, Makhema).

## Data Availability

The data that support the findings of this study are available from the corresponding author upon reasonable request.
